# Supplementary material for: VEGF Overexpression Significantly Increases Nanoparticle-Mediated siRNA Delivery and Target-Gene Downregulation
Source: Pharmaceutics. 2022 Jun 14;14(6):1260. doi: 10.3390/pharmaceutics14061260 (PMC9229257; doi:10.3390/pharmaceutics14061260)
Supplement: Supplementary file 1 [file pharmaceutics-14-01260-s001.zip › pharmaceutics-1706226-supplementary.pdf]

## Supplementary Information

**Figure S1:** $^1$H NMR spectrum of PEGylated PEI.
**Figure S2:** **(A)** TEM image of NPs and **(B)** histogram of the PEG-PEI NP size distribution.
**Figure S3:** DLS characterization of PEG-PEI NP hydrodynamic diameter and PdI.
**Figure S4:** Downregulation of Chkα mRNA in 231 WT cells under normoxic and hypoxic conditions.
**Figure S5:** Summary of *in vivo* and *ex vivo* Cy5.5 fluorescence images of 231 WT tumor bearing mice.
**Figure S6:** Summary of *in vivo* and *ex vivo* Cy5.5 fluorescence images of 231 VEGF tumor bearing mice.
**Figure S7:** Comparison of GPC, PC, Cho and total choline levels in untreated 231 WT and untreated 231 VEGF tumor tissue.

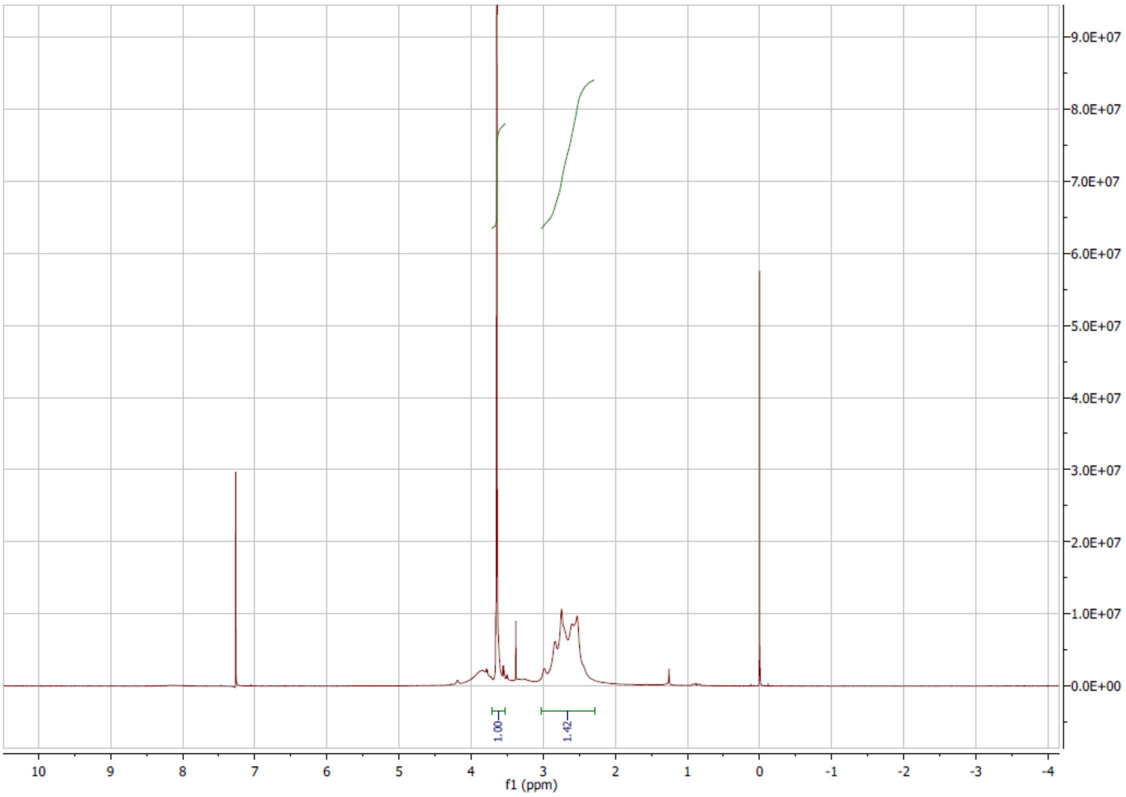


**Figure S1:** $^1$H NMR spectrum of PEGylated PEI in CDCl3: δ 3.60 (m, O$CH_2CH_2$O), 3.0-2.0 (m, N$CH_2CH_2$N))

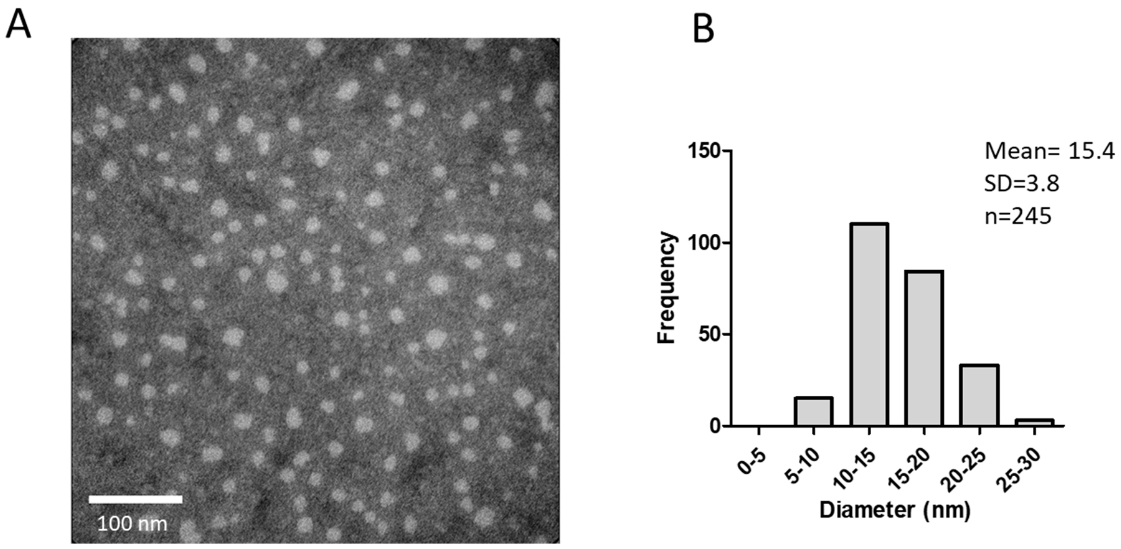


**Figure S2:** Transmission electron microscopy (TEM) image and histogram of the PEG-PEI NP size distribution. (A) TEM image of whole FOV under 150k magnification. 245 particles in one continuous region of interest were measured for diameter and graphed for particle size distribution. (B) Particle size distribution graph. Measured diameter frequency plotted against particle diameter range. The diameter range span is 5nm, mean diameter is 15.4nm, standard deviation is 3.8.

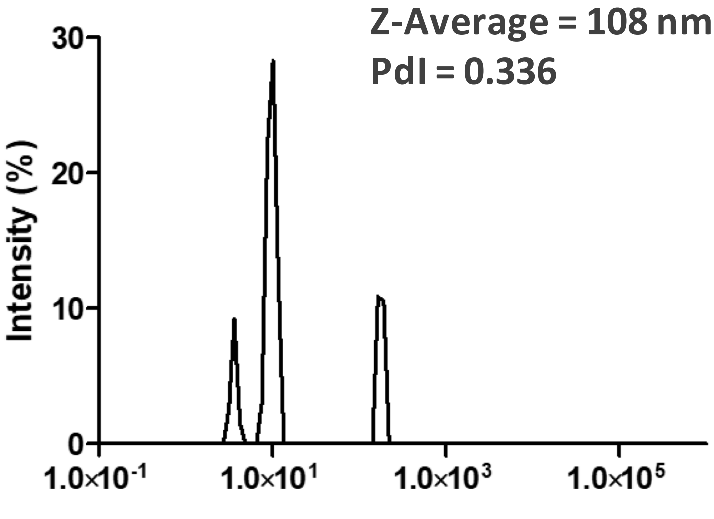


**Figure S3:** DLS characterization of PEG-PEI NP size distribution by intensity. A total of 2 measures, 10 runs per measurement were taken. Z-average is 108nm. The Poly-disperse Index (PdI) is 0.336.

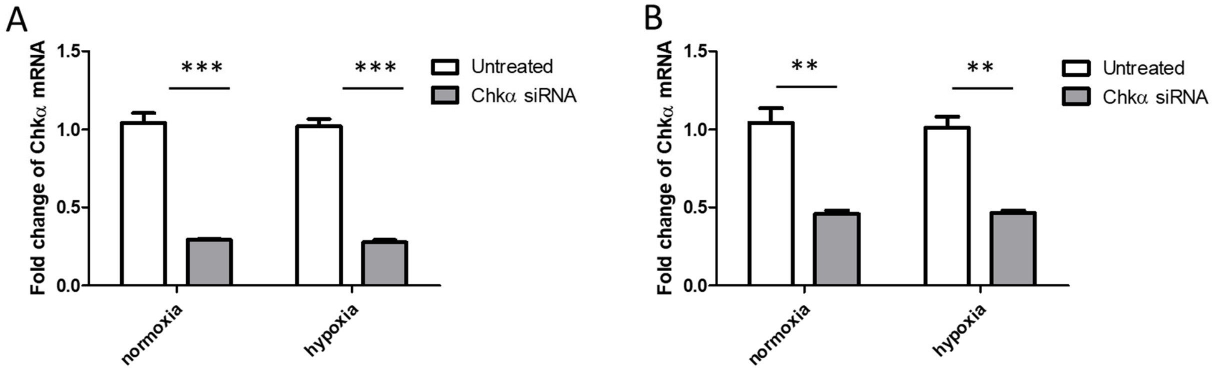


**Figure S4:** Downregulation of Chkα mRNA in 231 WT breast cancer cells under normoxic and hypoxic conditions. **A.** Fold change of Chkα mRNA in 231 WT cells under normoxia (0.29 ± 0.01) or hypoxia (0.27 ± 0.01) using the transfection agent D-FECT 4. **B.** Fold change of Chkα mRNA in 231 WT cells under normoxia (0.45 ± 0.02) or hypoxia (0.46 ± 0.04) with Chkα siRNA PEI NP transfection. Values represent Mean ± SEM from 3 experiments, ** $p < 0.01$, *** $p < 0.001$. Hypoxia did not alter the ability of siRNA to downregulate the target gene Chkα.

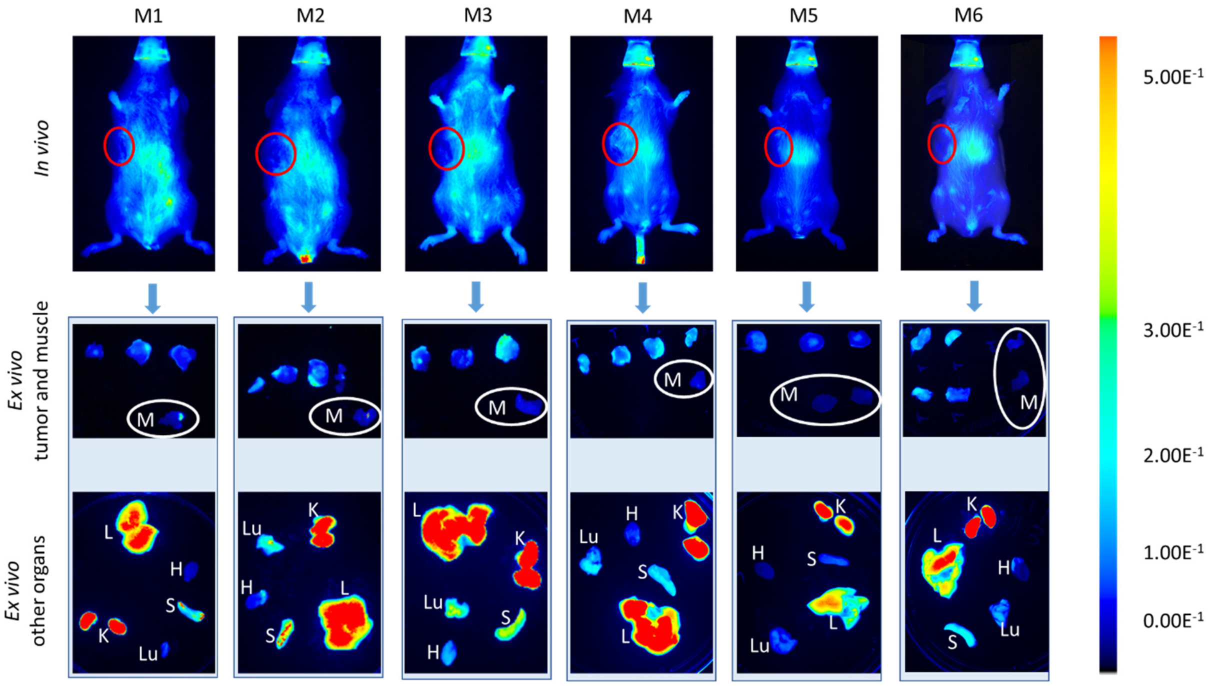


**Figure S5:** *In vivo* and *ex vivo* Cy5.5 fluorescence images of mice with 231 WT tumors imaged in the study. Top row: *In vivo* images of 231 WT mice with Chkα siRNA NP administration, red circled regions mark the tumor. Middle Row: *Ex vivo* images of 2 mm tumor slices (3-4 slices per tumor) and muscle fresh tissue slices (1-2 slices per mouse), white circles mark muscle tissue slices (M). Bottom Row: *Ex vivo* fluorescence images of the liver (L), heart (H), spleen (S), lung (Lu), and kidneys (K).

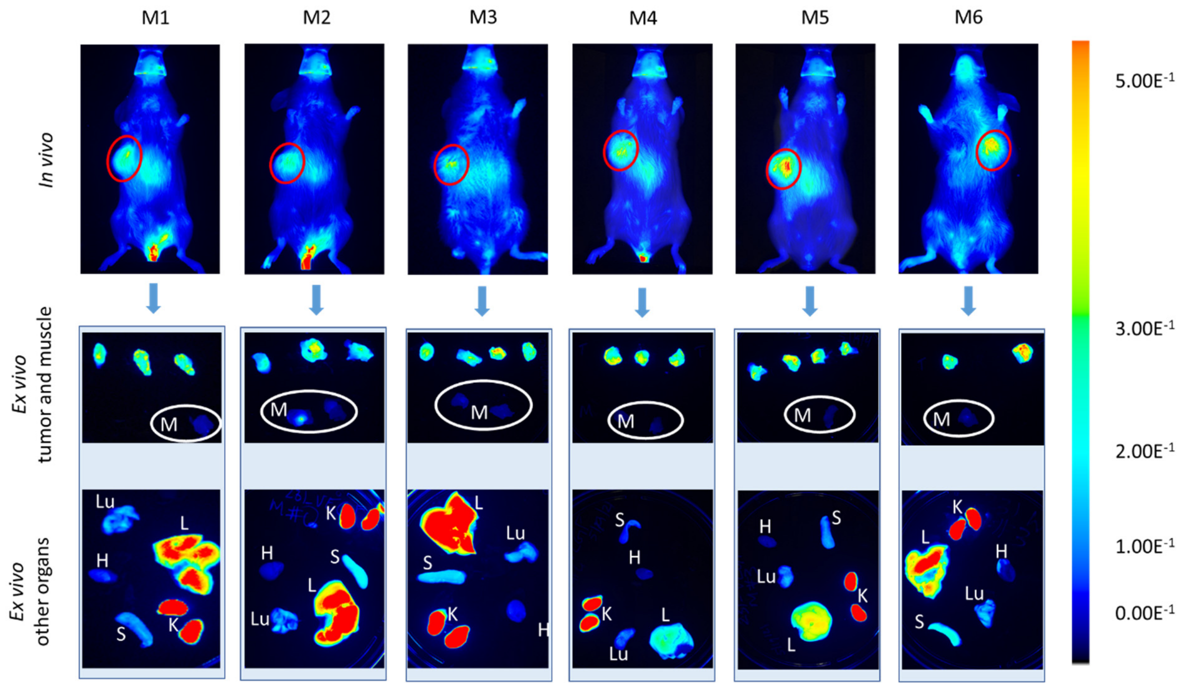


**Figure S6**: *In vivo* and *ex vivo* Cy5.5 fluorescence images of mice with 231 VEGF tumors imaged in the study. Top row: *In vivo* images of 231 VEGF mice with Chkα siRNA NP administration, red circled regions mark the tumor. Middle Row: *Ex vivo* images of 2 mm tumor slices (3-4 slices per tumor) and muscle fresh tissue slices (1-2 slices per mouse), white circles mark muscle tissue slices (M). Bottom Row: *Ex vivo* fluorescence images of the liver (L), heart (H), spleen (S), lung (Lu), and kidneys (K).

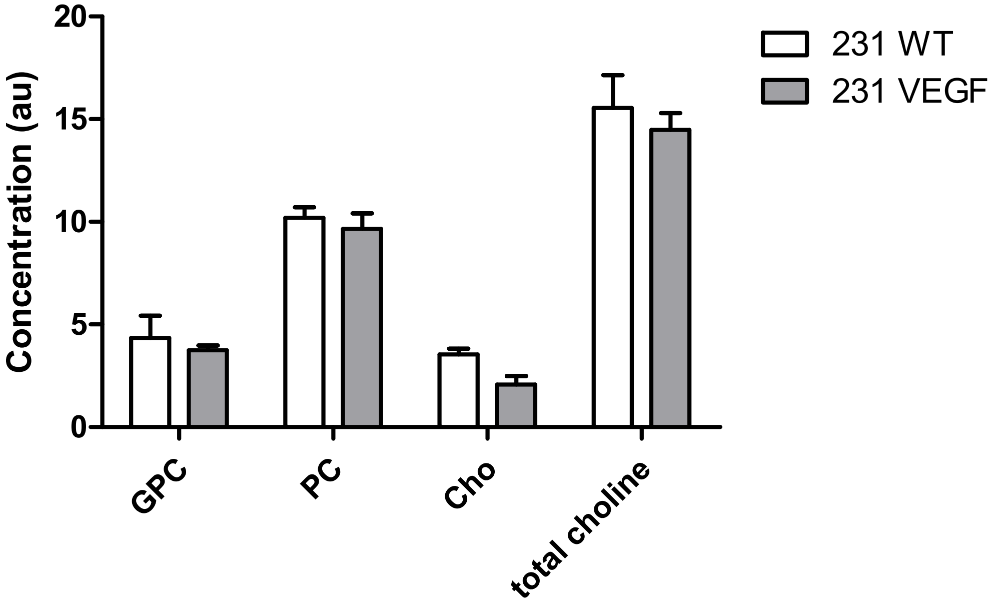


**Figure S7:** Comparison of GPC, PC, Cho, and total choline level in untreated 231 WT and 231 VEGF tumor extracts. No significant difference was observed in basal levels of choline metabolites between 231 WT and 231 VEGF tumors.
